# Association between urinary arsenic and hearing threshold shifts in adults in the United States, National Health and Nutrition Examination Survey, 2015–2016

**DOI:** 10.3389/fpubh.2024.1431122

**Published:** 2024-12-18

**Authors:** Lili Long, Zhenchao Jia, Tao Liu

**Affiliations:** ^1^Department of Otorhinolaryngology, Sichuan University Hospital of Sichuan University, Chengdu, China; ^2^Department of Prevention and Health Care, Sichuan University Hospital of Sichuan University, Chengdu, China; ^3^Department of Otorhinolaryngology, Air Force Hospital Medical Service Department in Western Theatre, Chengdu, China

**Keywords:** arsenic metabolites, hearing threshold shift, National Health and Nutrition Examination Survey, cross-sectional study, adults

## Abstract

**Background:**

Hearing loss (HL) is a common sensory disorder in humans. Studies on the relationship between arsenic, which is a highly toxic and widely distributed heavy metal with a health risk to humans, and hearing status in humans are contradictory and mostly focused on people living in arsenic-contaminated areas. This study investigated the association between urinary arsenic levels and hearing threshold shifts in the general population in the United States.

**Methods:**

Overall, 1,017 adults (aged 20–69 years) from the National Health and Nutrition Examination Survey (2015–2016) were included in this study. HL was defined as pure-tone average (PTA) ≥ 20 dB at frequencies 500, 1,000, 2000, and 4,000 Hz in the better-hearing ear. Total urinary arsenic (uAs) and dimethylarsinic acid (uDMA) levels were analyzed. Multivariate linear regression analyses and smooth curve fitting were performed to evaluate the correlations between uDMA, uAs, and low-, speech-, and high-frequency hearing levels.

**Results:**

The mean age of the participants was 42.13 ± 13.66 years, including weighted 48.67% men and 12.88% participants with sensorineural HL. After adjusting for potential confounders in the multivariate linear regression model, higher uDMA levels were significantly associated with poor low-, and speech-frequency PTAs, with no differences among participants by age or sex. Smooth curve fitting indicated a nonlinear relationship between uAs and high-frequency PTA hearing threshold shifts. The uAs levels were positively associated with high-frequency PTA until the turning point of 1.54 (adjusted *β* 4.53, 95% CI 1.16, 7.90; *p* = 0.0085), beyond which this association was not observed (adjusted β −0.43, 95% CI −1.57, 0.71; *p* = 0.4600).

**Conclusion:**

We found positive associations between urinary arsenic metabolites uDMA, uAs levels and poor hearing threshold shifts in US adults. This study provides new evidence for the association between arsenic exposure and auditory function.

## 1 Introduction

Hearing loss (HL) is one of the most common sensory disabilities in humans, affecting over 1.5 billion people globally. An estimated 430 million people have moderate or higher severity HL in the better-hearing ear. HL has lifelong ramifications, including adverse health and social and economic development, which can negatively affect the development of language and speech during childhood, education, communication, cognition, employment, and mental health. The annual global cost of HL is over $980 billion annually (1). To prevent and treat HL, extensive research has been devoted to investigating its common causes, such as noise exposure, aging, and ototoxic drug use; the role of exposure to environmental heavy metals in HL is especially concerning (2).

Arsenic is a highly toxic heavy metal. Natural sources such as mineral weathering, geothermal processes, and volcanic terrain lead to a massive distribution of arsenic in soil and water. Arsenic is widely used in anthropogenic activities such as mining, fossil fuel combustion, pesticides/herbicides, and wood timber and leather preservation (3, 4). Arsenic is found in different oxidation states and multiple inorganic and organic forms, which can be converted into each other in nature (5). Animals convert inorganic arsenite and arsenate into methylarsonic acid, dimethylarsinic acid, monomethylarsonous acid, and dimethylarsinous acid in a series of methylating reactions (5). Most arsenic is distributed to the kidneys, muscles, liver, nerve tissue, and mainly excreted from the mammalian body in the urine (5). Metabolic inhibition, oxidative stress, genotoxicity, and several epigenetic factors including histone modifications and micro-RNA are believed to be the underlying mechanisms of arsenic toxicity (5). The World Health Organization has recommended the maximum acceptable arsenic concentration in drinking water as 10 μg/L (10 ppb) (5). More than 200 million people worldwide are estimated to be exposed to unsafe levels of arsenic (5), which poses a health risk to humans. Previous studies have shown that human exposure to arsenic is associated with cancer of the kidney, lung, bladder, skin, prostate, and liver (6); cardiovascular disorders (7), neurological diseases, and complications in children (8) and adults (9); and diabetes mellitus (10), reproductive disorders, and developmental disorders (11).

Studies investigating the relationship between arsenic and HL are contradictory and have mostly focused on individuals living or working in arsenic-contaminated areas or those with acute high doses of arsenic poisoning (12–22). Bencko et al. (12) reported low-, and high-frequency HL in 56 10-year-old children residing near a power plant burning local coal of high arsenic content. Another study investigating 145 Bangladeshi living in areas of arsenic-polluted drinking water showed levels of arsenic in toenails were significantly correlated with HL at 4 kHz (13). Shokoohi et al. reported increased risk of HL in 120 cases exposed to arsenic in Iran (14). Sugiyama et al. reported that 70.5% of 190 cases had resided near an arsenic mine had HL, with difficulty to rule out the possibility of other factors, like age which may contribute to HL (15). Li et al. reported 48 cases drinking tube well water contaminated with arsenic had significantly higher risks of HL at 4 kHz (16). Investigations carried out on 13,021 people drinking arsenic contaminated water (50–1860 μg/L) in Inner Mongolia, China showed 5.88% of HL (17). De Capitani et al. reported a case of severe HL in a young male due to acute poisoning from pesticide monosodium methanarsenate containing organic arsenicals (18). However, Milham et al. (19) investigated the hearing of 7 children with high average urinary arsenic levels, who were living near a copper smelter, and found their hearing were within normal limits. Supapong et al. (20) reported 27 females drinking water from shallow wells which had a low level of arsenic showing no difference in brainstem auditory evoked potentials compared with the referent group. A study of 121 workers exposed to arsenic in Taiwan, China showed no relationship between arsenic exposure and hearing thresholds (21). Another study showed no significant correlation between arsenic concentrations and hearing parameters in 59 artisanal gold miners in Nicaragua whose nail arsenic concentrations were far exceeded reference levels (22). Only two studies have reported the effect of arsenic on hearing in the general population (2, 23). One study showed no overall association between quartiles of urinary arsenic levels and HL in US adolescents (2). Another study showed no evidence of association between urinary arsenic level and hearing disturbance in US adults (23). However, self-reported hearing conditions instead of pure tone audiometry data were used in the analysis. Here, we performed a large-scale cross-sectional study to investigate the relationship between urinary arsenic and hearing levels in adults aged 20–69 years who participated in the United States (US) National Health and Nutrition Examination Survey (NHANES) 2015–2016, which is the most recent survey cycle.

## 2 Methods

### 2.1 Study design and population

The NHANES is a nationwide cross-sectional survey conducted by the US Center for Disease Control and Prevention that collects health-related representative data of US non-institutional civilians on a 2-year basis; this consists of in-person interviews, physical examinations, and laboratory test results using a stratified multistage probability sampling design. All NHANES data analyzed in this study are publicly available on the NHANES official website.^1^ The National Center for Health Statistics Research Ethics Review Board approved the NHANES, and all participants provided written informed consent.

**Figure 1:**
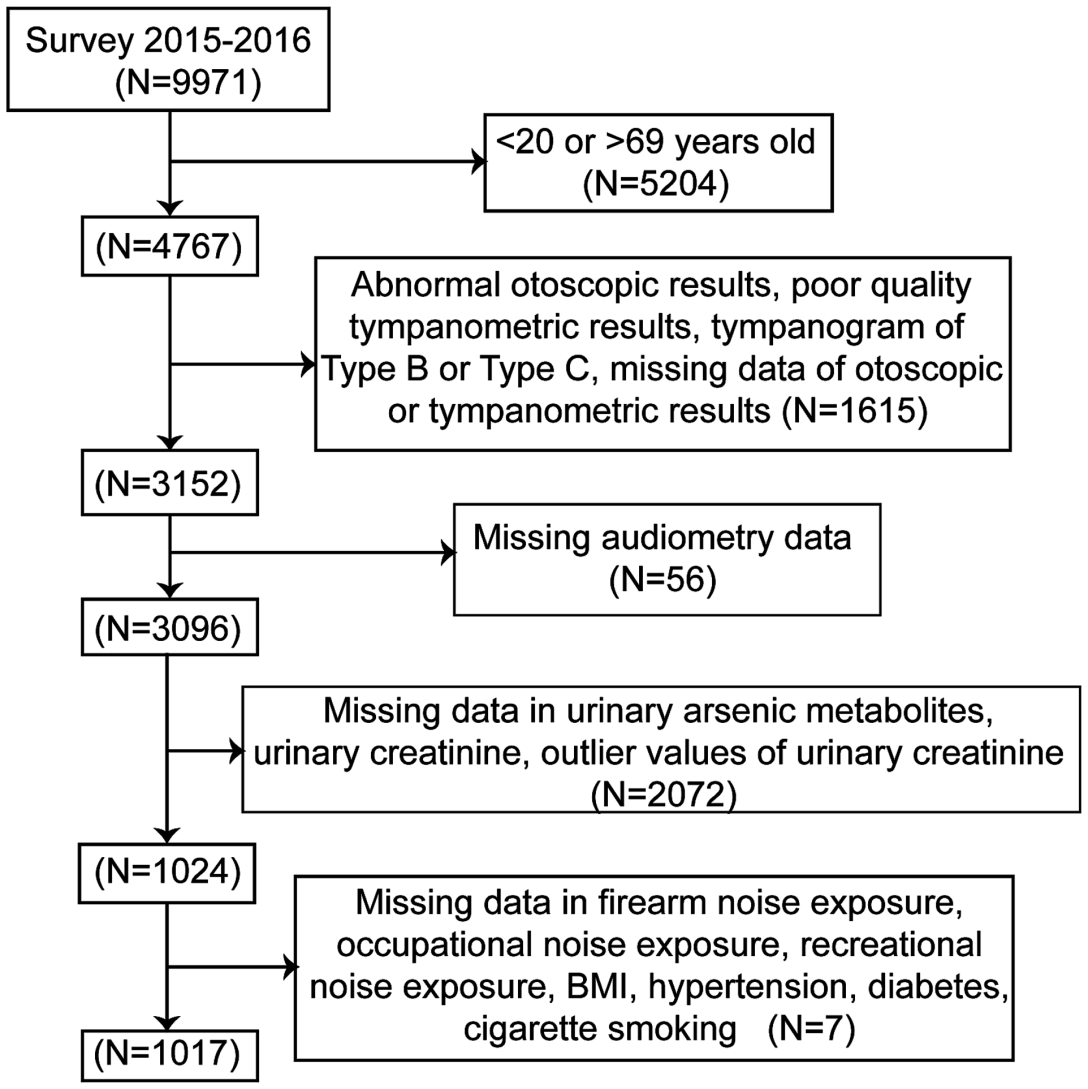
Flow chart of the selection process. NHANES, National Health and Nutrition Examination Survey.

Participants in the NHANES cycle 2015–2016, which contained test data for both urinary arsenic metabolites and audiometric examinations of adults aged 20–69 years were included in this study. A total of 1,017 individuals were included in the final analysis. The participants with abnormal otoscopic examination results, poor-quality tympanogram results or tympanogram of Type B or Type C were excluded to avoid analyzing conductive or mixed HL data. Figure 1 illustrates the selection procedure used in this study.

### 2.2 Urinary arsenic measures

Urine is analyzed because arsenic is mainly excreted from the mammalian body in the urine. Urine samples were collected from all examined participants aged 3–5 years and those aged ≥6 years from a one-third subsample. Vials holding urine samples were stored under appropriate frozen (−30°C) conditions until shipped to National Center for Environmental Health, Center for Disease Control and Prevention for testing. Concentrations of arsenic metabolites were measured using inductively coupled-plasma dynamic reaction cell-mass spectrometry (ICP-DRC-MS), which can achieve rapid and accurate quantification of total urinary arsenic. The NHANES quality assurance and quality control (QA/QC) protocols meet the 1988 Clinical Laboratory Improvement Act mandates. Several techniques were used to monitor laboratory team performance. Details are available on the NHANES official website.^2^ Arsenic metabolites with a detection rate ≥ 60% in the samples were selected for analysis in our study including total urinary arsenic (uAs) and urinary dimethylarsinic acid (uDMA). Other arsenic compounds with a detection rate of <60% in the urine, such as arsenous acid, arsenic acid, arsenobetaine, arsenocholine, and monomethylarsonic acid, were excluded. The limit of detection divided by the square root of two was used for any sample below the limit of detection. The uAs concentrations were ln-transformed to normalize the skewed distribution before the analysis (23, 24). The ln-transformed creatinine-adjusted uAs concentrations were not used because the data were skewed.

### 2.3 Audiometric measurement

Audiometric measurements were performed by trained examiners at the mobile examination center. Participants aged 20–69 years were included in the study. An otoscopic examination of the ear canals and eardrums was conducted for excessive ear cerumen and other physical abnormalities. Tympanometry was performed using a tympanometer (Interacoustics Model Titan, Denmark to identify middle ear pathologies that may contribute to conductive HL). An audiometer (Interacoustics Model AD226, Denmark) was used to measure the pure-tone air-conduction audiometry of both ears. Participants who were unable to remove their hearing aids or could not tolerate the headphone-induced ear pain were excluded. The complete detailed procedure is available on the website.^3^

In this study, the low-frequency pure-tone average (PTA) was defined as a PTA of 0.5, 1, and 2 kHz; speech-frequency PTA as a PTA of 0.5, 1, 2, and 4 kHz; and high-frequency PTA as a PTA of 4, 6, and 8 kHz in the better-hearing ear. The World Health Organization (WHO) criteria 2021 defined HL as speech-frequency PTA ≥ 20 dB in the better-hearing ear (1).

### 2.4 Potential covariates

Age, sex, race, educational level, body mass index (BMI), hypertension, diabetes, cigarette smoking, firearm noise exposure, occupational noise exposure, and recreational noise exposure were included as potential covariates. Information on demographic and potential hearing-related covariates, including age, sex, race, educational level, cigarette smoking, firearm noise exposure, occupational noise exposure, and recreational noise exposure, was obtained through home-based interviews. BMI was calculated from the weight and height data of the participants, which were obtained during physical examinations at a Mobile Examination Center.

Self-reported hypertension was defined as a “Yes” response to the question, “Have you ever been told by a doctor or other health professional that you had hypertension, also called high blood pressure?” or participant-reported treatment for high blood pressure. Hypertension was defined as a mean blood pressure (from three measurements) ≥ 130 mm Hg for systolic blood pressure or ≥ 80 mm Hg for diastolic blood pressure (25). Participants were considered to have diabetes if they met any of the following conditions: fasting plasma glucose ≥126 mg/dL, oral glucose tolerance test ≥200 mg/dL, glycated hemoglobin ≥6.5%, self-reported diabetes, and currently on insulin or oral medication use (26). Participants were categorized as never, former, or current smokers from the question, “Have you smoked at least 100 cigarettes in your entire life?” and “Do you now smoke cigarettes?” (27). Firearm noise exposure was defined as “ever used firearm for any reason” (27). Occupational noise exposure was defined as “ever had a job exposure to loud noise for ≥4 h a day, several days a week” (27). Recreational noise exposure was defined as “outside of a job, ever been exposed to very loud noise or music for ≥10 h a week” (27).

### 2.5 Statistical analysis

This study used subsample A weights (WTSA2YR) of 2015–2016 NHANES cycle to estimate representative measures for the civilian non-institutionalized US population, following the analytic guidelines of the National Center for Health Statistics (28). uAs and uDMA levels were Ln-transformed and classified into tertiles. The weighted demographic characteristics of the study participants grouped by the tertiles of uAs were evaluated. Data are reported as numbers and percentages for categorical variables or as mean ± standard deviation (SD) for continuous variables. Regression coefficients (*β*) and 95% confidence intervals (CIs) were generated using multivariate linear regression models to test the associations between uDMA, uAs and hearing threshold shifts, adjusting for potential covariables, including age, sex, race, education level, BMI, hypertension, diabetes, cigarette smoking status, and noise exposure. Ln-transformed uDMA and uAs levels were converted from continuous variables to categorical variables (tertiles), and the tertiles were modeled as continuous variables to test the linear trend. The interactions of age and sex with uDMA and uAs in influencing hearing thresholds were also tested. Multivariate analyses stratified according to age and sex were performed.

**Figure 2:**
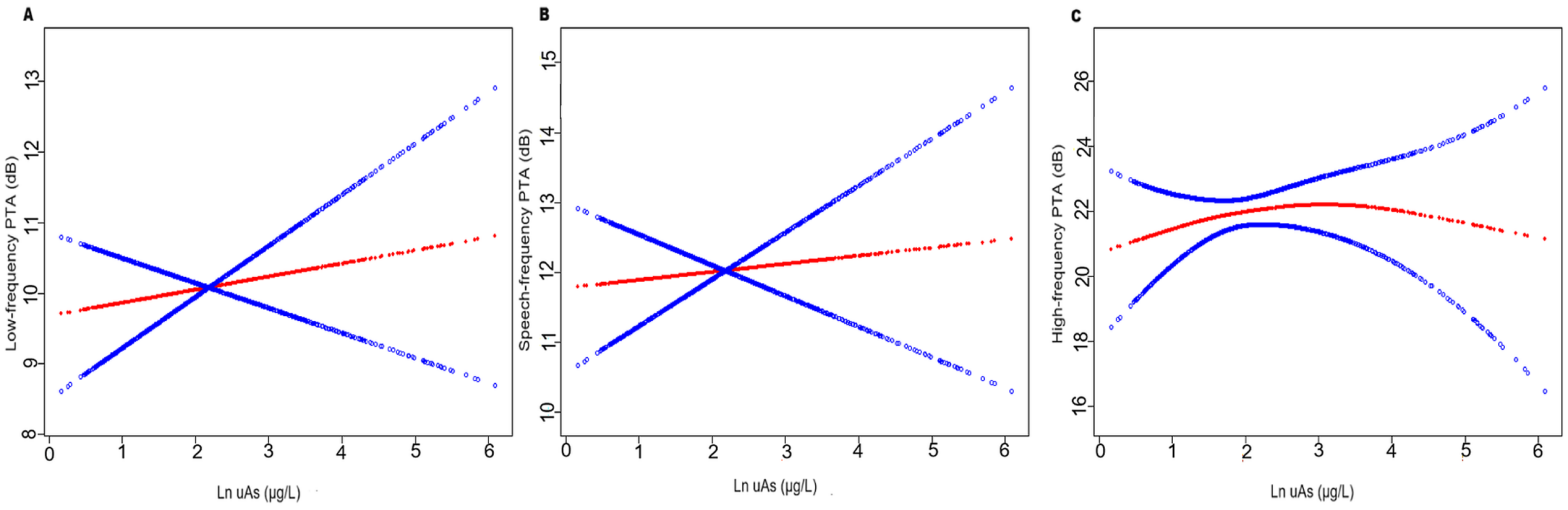
The relationship between uAs levels and hearing threshold shifts (*N* = 1,017): **(A)** low-frequency pure-tone average (PTA), **(B)** speech-frequency PTA and **(C)** high-frequency PTA. The red line represents the smooth curve fit between variables. In comparison, blue bands represent the 95% CI. Age, gender, race, education level, BMI, hypertension, diabetes, cigarette smoking, firearm noise exposure, occupational noise exposure and recreational noise exposure were adjusted.

Smoothed curves were fitted using R to explore the relationship between uDMA, uAs, and hearing threshold shifts after adjusting for potential covariates (Figure 2). A weighted two-piecewise linear regression model with a single inflection point was used to examine the effect of uDMA and uAs on hearing threshold shifts, shown as a smoothing plot. A significant log-likelihood ratio test was performed to determine the presence of a threshold. The inflection point was calculated using a recursive algorithm and a maximum-likelihood model. All statistical tests were conducted using R 3.6.1 and R 4.3.1. Statistical *p* value was set at *p* < 0.05.

## 3 Results

### 3.1 Baseline characteristics

The characteristics of participants according to uAs tertiles are shown in Table 1, and a total of 1,017 participants aged 20–69 years who had measured data for hearing and urinary arsenic levels were included in this study. 45.77% of the participants were men, and the mean age was 42.13 ± 13.66 years old. Overall, 18.43% of adults had sensorineural HL. The ranges of uAs tertiles 1–3 were < 4.17, 4.17–10.72 and > 10.72 μg/L, respectively. Compared with the lowest tertile, participants in the tertile 3 group tend to had poorer speech- and high-frequency hearing levels, non-Hispanic black ethnicity and other races (all *p* < 0.05).

**Table 1:** The weighted demographic characteristics of study participants by uAs.

Variables	Total	Tertile 1 (*N* = 339)	Tertile 2 (*N* = 339)	Tertile 3 (*N* = 339)	*P* ^a^
Age, years	42.13 ± 13.66	41.23 ± 13.84	42.20 ± 12.64	43.27 ± 13.33	0.1526
Low-frequency PTA, dB^b^	9.94 ± 10.20	9.29 ± 10.98	9.78 ± 8.98	11.03 ± 10.26	0.0822
Speech-frequency PTA, dB^b^	11.97 ± 11.37	11.04 ± 11.83	11.86 ± 10.84	13.39 ± 11.16	**0.0266**
High-frequency PTA, dB^b^	22.28 ± 18.82	20.91 ± 17.68	21.94 ± 20.20	24.57 ± 18.52	**0.0380**
BMI, kg/m2	29.69 ± 7.21	29.51 ± 7.39	29.98 ± 7.09	29.62 ± 7.08	0.6771
Male, %	45.77	42.11	50.01	46.08	0.1032
Race, %					**<0.0001**
Mexican American	9.83	8.58	13.49	7.46	
Non-Hispanic White	62.11	73.58	56.65	52.34	
Non-Hispanic Black	11.37	7.22	12.26	16.12	
Other races	16.69	10.62	17.60	24.09	
Education level, %					0.4594
Below high school	12.82	11.15	12.36	15.68	
High school	18.31	19.01	17.29	18.49	
Above high school	68.86	69.84	70.35	65.83	
Hypertension, %	40.54	37.36	44.56	40.43	0.1439
Diabetes, %	13.29	10.82	14.01	15.89	0.1376
Cigarette smoking, %					0.7388
Never smoker	58.13	59.14	59.37	55.31	
Former smoker	24.23	22.67	24.41	26.19	
Current smoker	17.65	18.19	16.22	18.51	
Firearm noise, %	52.92	56.31	50.87	50.53	0.2166
Occupational noise, %	29.99	30.81	30.83	27.92	0.6607
Recreational noise, %	14.05	14.15	14.34	13.59	0.9630
^c^Hearing loss, %	18.43	15.99	16.97	23.44	**0.0321**

NHANES 2015–2016 cycle, *N* = 1,017. PTA pure-tone average, BMI body mass index.

a *p values of continuous variables and categorical variables were calculated by weighted linear regression model and weighted chi-square test, respectively.*

b Low-, speech-, high-frequency PTA values in the better ear were computed from the average of hearing thresholds of 0.5, 1 and 2 kHz, 0.5, 1, 2 and 4 kHz, 4, 6 and 8 kHz, respectively.

c Earing loss was defined as the PTA value ≥ 20 dB in the better ear. Significant values are in bold.

### 3.2 The association of arsenic metabolites with hearing levels

We have used two multivariate linear regression models to show the relationship between arsenic metabolites uDMA and uAs levels with low-, speech-, and high-frequency hearing levels in Table 2: crude model, no covariate was adjusted; adjusted model, age, gender, race, education level, BMI, hypertension, diabetes, cigarette smoking, firearm noise exposure, occupational noise exposure, recreational noise exposure were adjusted. We found a significantly positive association between uDMA levels and low-, and speech-frequency PTA hearing threshold shifts in both the unadjusted model (crude model) and adjusted model. Individuals in the tertile 3 group (uDMA >5.63 μg/L) had worse low-, and speech-frequency PTAs compared with those in the tertile 1 group (uDMA <2.22 μg/L). Regarding uAs, although we found a significantly positive association between uAs levels with hearing levels of all frequencies in the crude model, no significant associations were found for the adjusted model.

**Table 2:** Multivariable linear regression models assessing the relationship between uDMA, uAs levels and PTA hearing thresholds (*N* = 1,017).

		Ln uDMA (μg/L)			*P*_trend_
		Tertile 1 (*N* = 339)	Tertile 2 (*N* = 337)	Tertile 3 (*N* = 341)	
Low-frequency PTA	Crude βs	Ref	0.49 (−0.98, 1.96)	2.42 (0.86, 3.97)	**0.0033**
	Adjusted βs	Ref	−0.12 (−1.47, 1.23)	2.29 (0.83, 3.74)	**0.0040**
Speech-frequency PTA	Crude βs	Ref	1.02 (−0.62, 2.66)	2.73 (0.99, 4.46)	**0.0023**
	Adjusted βs	Ref	−0.07 (−1.46, 1.33)	2.38 (0.88, 3.88)	**0.0036**
High-frequency PTA	Crude βs	Ref	1.09 (−1.63, 3.81)	2.30 (−0.58, 5.19)	0.1163
	Adjusted βs	Ref	−1.35 (−3.36, 0.65)	1.41 (−0.74, 3.57)	0.2920

		Ln uAs (μg/L)			*P*_trend_
		Tertile 1 (*N* = 339)	Tertile 2 (*N* = 339)	Tertile 3 (*N* = 339)	
Low-frequency PTA	Crude βs	Ref	0.49 (−1.00, 1.98)	1.74 (0.20, 3.28)	**0.0302**
	Adjusted βs	Ref	0.18 (−1.19, 1.55)	1.08 (−0.36, 2.51)	0.1528
Speech-frequency PTA	Crude βs	Ref	0.83 (−0.83, 2.48)	2.35 (0.64, 4.07)	**0.0079**
	Adjusted βs	Ref	0.25 (−1.17, 1.66)	1.41 (−0.07, 2.89)	0.0704
High-frequency PTA	Crude βs	Ref	1.03 (−1.71, 3.77)	3.66 (0.82, 6.50)	**0.0132**
	Adjusted βs	Ref	−0.18 (−2.21, 1.85)	1.68 (−0.44, 3.80)	0.1432

Crude Model = unadjusted. Adjusted Model = Crude Model + age, gender, race, education level, BMI, hypertension, diabetes, cigarette smoking, firearm noise exposure, occupational noise exposure, recreational noise exposure; PTA, pure tone average; CI, confidence interval.

Significant values are in bold.

Supplementary Tables S1 S2 showed no significant association of both uDMA and uAs levels with hearing levels in the subgroup analysis stratified by age and sex, respectively.

### 3.3 Analyses of the non-linear relationship between arsenic metabolites and hearing levels

The relationship between uAs and high-frequency PTA was found to be nonlinear rather than linear (*P* for nonlinearity = 0.014, Table 3). The non-linearity relationship between uAs levels and high-frequency PTA indicated an inverted U-shaped pattern (Figure 2). Using a two-part piecewise linear regression model with all confounders being adjusted, the high-frequency PTA increased with increasing uAs levels until the turning point of 1.54, whereas it did not as uAs exceeding 1.54 (*β* = 4.53; 95% CI: 1.16, 7.90; *p* = 0.0085) (Table 3). No nonlinear relationship was observed between uDMA and hearing threshold shifts (Supplementary Tables S3).

**Table 3:** The results of two-piecewise linear regression model between uAs levels and hearing thresholds (*N* = 1,017).

Exposure variables	Low-frequency PTA	Speech-frequency PTA	High-frequency PTA
Cut off point of uAs	3.86	2.56	1.54
< Cut off point of uAs	0.76 (0.04, 1.48), **0.0380**	1.36 (0.31, 2.41), **0.0110**	4.53 (1.16, 7.90), **0.0085**
≥ Cut off point of uAs	−1.03 (−3.85, 1.79), 0.4752	−0.56 (−1.82, 0.70), 0.3866	−0.43 (−1.57, 0.71), 0.4600
Loglikelihood ratio test	0.263	0.052	**0.014**

Significant values are in bold.

## 4 Discussion

In this nationwide cross-sectional study, we explored, for the first time, the relationship between uAs levels and low-, speech-, and high-frequency hearing threshold shifts among adults aged 20–69 years, using the NHANES 2015–2016 database.

Our study found a significant linear association between uDMA levels and poor low-, and speech-frequency PTAs, with no significant interactions of uDMA level with age or sex. Additionally, a significant non-linear association was observed between the uAs levels and poor high-frequency PTA in adults in the United States. These results are consistent with those of previous studies (12, 13, 15–18). Bencko et al. reported that arsenic-exposed children living near a power plant that burned high arsenic-containing coal in Czechoslovakia had significantly worse pure-tone thresholds than non-exposed children at 125, 250, and 8,000 Hz (low and high frequencies) (12). Guo et al. reported a higher prevalence of hearing impairment in adults in counties with drinking water contaminated with naturally occurring arsenic in inner Mongolia, China (17). Xiang et al. reported that individuals aged 12–29 years who had been drinking arsenic-contaminated tube well water in Bangladesh had a significantly higher risk of hearing impairment at 4 kHz than those in the control group, showing that oral exposure to arsenic harms hearing in young adults (16). The same research group also found that the levels of arsenic in toenails, but not in urine, were significantly correlated with HL at 4, 8, and 12 kHz in 145 Bangladeshi individuals aged 12–55 years (13). A descriptive study by Sugiyama et al. reported that 70.5% of residents near an arsenic mine in Toroku, Japan, experienced deafness 45 years after the initial arsenic exposure (15). A case report by De Capitani et al. showed a severe bilateral neurosensory pattern of deafness with symmetrical losses of 110–120 dB at all tested frequencies in both ears of a 36-year-old man who had attempted suicide by ingesting approximately 250 mL of monosodium methanarsenate, a pesticide containing synthetic forms of organic arsenicals (18). Auditory brainstem-evoked potentials were absent bilaterally after maximum stimulation (105 dB), confirming severe cochlear lesions in both ears (18).

In addition to investigations concerning hearing impairment in individuals living or working in arsenic–polluted areas or those with acute arsenic poisoning, the results of our large-scale research imply a harmful effect of environmental arsenic exposure on the hearing system in the general population in the US. In many countries worldwide, including the US, India, Bangladesh, China, and Mexico, a high level of inorganic arsenic is naturally present in groundwater (29). Almost half of the world’s population uses groundwater for domestic needs (30). In addition to groundwater, arsenic is found in common dietary sources including rice, grains, juices, and seafood (5, 31). uDMA, an inorganic arsenic, and total arsenic uAs were detected in more than 60% of samples. Arsenic can impair low- and high-frequency hearing in individuals without them being aware because of its relatively low extent and lack of influence on speech-fluency PTA (23). The harmful effects of arsenic on human hearing are concerning. The results of our study were contrary to those of previous studies that showed no relationship between arsenic levels and human hearing (2, 14, 19–23). Variations in the sample size, study design, analytical methodology, control for different covariates, and racial heterogeneity may explain the disparities between our results and those of previous studies.

Although several human-based association studies have revealed a link between arsenic exposure and hearing status, the mechanistic impact of arsenic exposure on the underlying auditory pathogenesis remains poorly understood. An animal experiment showed that mice exposed to arsenic ex vivo exhibited significantly decreased numbers of auditory neurons and fibers (16). Other experiments observed extravasates in different parts of the middle and inner ear, inner ear cavities filled with a serous or serofibrinous transudate, and degeneration of the cells in the spiral and vestibular ganglion (32). Oxidative stress, metabolic inhibition, genotoxicity, and epigenetic alterations, including micro-RNA-dependent regulation, are some of the molecular mechanisms underlying arsenic toxicity (5).

This study was conducted using large-scale population-based representative data with adjustments for critical confounders and rigorous quality control during data collection to establish a relationship between urinary arsenic concentrations and hearing impairment. However, this study had a few limitations. First, interpretation of our findings is strongly limited by the cross-sectional design of this study. The temporality of the association between exposure and outcomes could not be confirmed, so causation could not be established from our study. As with all cross-sectional human studies, residual confounding and confounding by unmeasured variables cannot rule out. There are some potential confounders we could have wished to control but were unable to calculate in our study, like dietary intake of all the ototoxic drugs, history of ear infection, congenital hearing impairment, chlorinated pollutants, et al. Prospective and Mendelian randomization studies are required to demonstrate the association between arsenic exposure and hearing levels definitively. Second, only urine sample data were used in the analysis. Nail and blood sample data can be compared to determine which is more sensitive in reflecting the relationship between arsenic exposure and hearing levels. Third, the data pertained only to US adults; caution should be exercised when generalizing the findings to people from other countries and races. Furthermore, additional basic studies are required to elucidate the effects of arsenic on auditory pathogenesis.

## 5 Conclusion

The results of the NHANES data analyses revealed a significant linear association between uDMA levels and poor low-, and speech-frequency PTAs, with no difference among participants by age or sex. Additionally, a significant non-linear association was observed between uAs levels and poor high-frequency PTA in US adults. Exposure to arsenic in the general population may harm the hearing system. Further research is essential to verify our results given the cross-sectional nature of the NHANES.

## Data Availability

The datasets presented in this study can be found in online repositories. The names of the repository/repositories and accession number(s) can be found in the article/Supplementary material.

## Footnotes

^1^ https://wwwn.cdc.gov/nchs/nhanes/continuousnhanes/default.aspx?BeginYear=2015

^2^ https://wwwn.cdc.gov/Nchs/Nhanes/2015-2016/UTAS_I.htm

^3^ https://wwwn.cdc.gov/Nchs/Nhanes/2015-2016/AUX_I.htm
