# Supplementary material for: Association between urinary arsenic and hearing threshold shifts in adults in the United States, National Health and Nutrition Examination Survey, 2015–2016
Source: Front Public Health. 2024 Dec 18;12:1431122. doi: 10.3389/fpubh.2024.1431122 (PMC11688340; doi:10.3389/fpubh.2024.1431122)
Supplement: Supplementary file 1 [file Table_1.docx]

Table S1 Adjusted^a^ associations between uDMA, uAs levels and hearing threshold shifts stratified by age (N=1017).

|  | Age, years | Ln uDMA (μg/L) β (95% CI) | | | *P*_trend_ | *P*_interaction_ |
| --- | --- | --- | --- | --- | --- | --- |
|  |  | Tertile 1 | Tertile 2 | Tertile 3 |  |  |
| **Low-frequency PTA** | ≥20, <40 (N = 460) | Ref | 0.49 (-0.77, 1.76) | 1.70 (0.40, 3.01) | **0.0123** | 0.9989 |
|  | ≥40, <60 (N = 400) | Ref | -1.27 (-3.91, 1.36) | 2.93 (-0.03, 5.89) | 0.0932 |  |
|  | ≥60, <69 (N = 157) | Ref | 2.23 (-2.16, 6.61) | 2.80 (-1.89, 7.48) | 0.2294 |  |
| **Speech-frequency PTA** | ≥20, <40 (N = 460) | Ref | 0.81 (-0.52, 2.13) | 1.56 (0.19, 2.93) | **0.0248** | 0.8980 |
|  | ≥40, <60 (N = 400) | Ref | -0.91 (-3.55, 1.73) | 3.04 (0.07, 6.01) | 0.0752 |  |
|  | ≥60, <69 (N = 157) | Ref | -0.05 (-4.83, 4.72) | 2.69 (-2.41, 7.79) | 0.3234 |  |
| **High-frequency PTA** | ≥20, <40 (N = 460) | Ref | 1.03 (-0.90, 2.97) | -0.06 (-2.06, 1.94) | 0.9667 | 0.2551 |
|  | ≥40, <60 (N = 400) | Ref | -1.41 (-4.59, 1.77) | 2.87 (-0.71, 6.44) | 0.1768 |  |
|  | ≥60, <69 (N = 157) | Ref | -10.42 (-18.71, -2.13) | -1.02 (-9.87, 7.84) | 0.6825 |  |
|  |  | Ln uAs (μg/L) β (95% CI) | | | *P*_trend_ | *P*_interaction_ |
|  |  | Tertile 1 | Tertile 2 | Tertile 3 |  |  |
| **Low-frequency PTA** | ≥20, <40 (N = 460) | Ref | 0.17 (-1.09, 1.43) | 1.09 (-0.24, 2.42) | 0.1226 | 0.9777 |
|  | ≥40, <60 (N = 400) | Ref | -0.66 (-3.49, 2.17) | 0.40 (-2.42, 3.23) | 0.7903 |  |
|  | ≥60, <69 (N = 157) | Ref | 4.21 (-0.01, 8.42) | 3.45 (-1.22, 8.11) | 0.1149 |  |
| **Speech-frequency PTA** | ≥20, <40 (N = 460) | Ref | 0.05 (-1.27, 1.36) | 0.93 (-0.46, 2.32) | 0.2153 | 0.6622 |
|  | ≥40, <60 (N = 400) | Ref | -0.49 (-3.32, 2.33) | 1.26 (-1.56, 4.09) | 0.3908 |  |
|  | ≥60, <69 (N = 157) | Ref | 3.02 (-1.61, 7.64) | 2.22 (-2.90, 7.34) | 0.3445 |  |
| **High-frequency PTA** | ≥20, <40 (N = 460) | Ref | -0.86 (-2.77, 1.06) | 0.40 (-1.63, 2.43) | 0.7988 | **0.0027** |
|  | ≥40, <60 (N = 400) | Ref | -1.19 (-4.56, 2.19) | 3.31 (-0.06, 6.68) | 0.0604 |  |
|  | ≥60, <69 (N = 157) | Ref | 1.09 (-7.13, 9.30) | -2.79 (-11.89, 6.31) | 0.5925 |  |

^a^ Adjusted for age, gender, race, education level, BMI, hypertension, diabetes, cigarette smoking, firearm noise exposure, occupational noise exposure, recreational noise exposure.
