# Supplementary material for: Association between urinary arsenic and hearing threshold shifts in adults in the United States, National Health and Nutrition Examination Survey, 2015–2016
Source: Front Public Health. 2024 Dec 18;12:1431122. doi: 10.3389/fpubh.2024.1431122 (PMC11688340; doi:10.3389/fpubh.2024.1431122)
Supplement: Supplementary file 2 [file Table_2.docx]

Table S2 Adjusted^a^ associations between uDMA, uAs levels and hearing threshold shifts stratified by gender (N=1017).

|  | Gender | Ln uDMA (μg/L) β (95% CI) | | | *P*_trend_ | *P*_interaction_ |
| --- | --- | --- | --- | --- | --- | --- |
|  |  | Tertile 1 | Tertile 2 | Tertile 3 |  |  |
| **Low-frequency PTA** | Female (N = 553) | Ref | 0.22 (-1.81, 2.25) | 2.79 (0.57, 5.02) | **0.0221** | 0.6120 |
|  | Male (N = 464) | Ref | -0.99 (-2.73, 0.75) | 1.32 (-0.54, 3.17) | 0.2012 |  |
| **Speech-frequency PTA** | Female (N = 553) | Ref | 0.66 (-1.34, 2.65) | 2.64 (0.45, 4.83) | **0.0226** | 0.6455 |
|  | Male (N = 464) | Ref | -1.35 (-3.29, 0.59) | 1.67 (-0.40, 3.75) | 0.1473 |  |
| **High-frequency PTA** | Female (N = 553) | Ref | 0.15 (-2.07, 2.37) | 0.40 (-2.03, 2.83) | 0.7503 | 0.6782 |
|  | Male (N = 464) | Ref | -3.43 (-6.80, -0.05) | 2.36 (-1.24, 5.96) | 0.2609 |  |
|  |  | Ln uAs (μg/L) β (95% CI) | | | *P*_trend_ | *P*_interaction_ |
|  |  | Tertile 1 | Tertile 2 | Tertile 3 |  |  |
| **Low-frequency PTA** | Female (N = 553) | Ref | -0.27 (-2.36, 1.83) | 2.14 (0.02, 4.26) | 0.0656 | 0.5241 |
|  | Male (N = 464) | Ref | -0.03 (-1.79, 1.74) | -0.19 (-2.06, 1.67) | 0.8427 |  |
| **Speech-frequency PTA** | Female (N = 553) | Ref | -0.21 (-2.27, 1.84) | 2.31 (0.23, 4.39) | **0.0424** | 0.5681 |
|  | Male (N = 464) | Ref | -0.06 (-2.04, 1.92) | 0.31 (-1.78, 2.39) | 0.7845 |  |
| **High-frequency PTA** | Female (N = 553) | Ref | -1.02 (-3.30, 1.25) | 2.29 (-0.01, 4.59) | 0.0813 | 0.5658 |
|  | Male (N = 464) | Ref | -0.02 (-3.46, 3.42) | 0.97 (-2.66, 4.61) | 0.6122 |  |

^a^ Adjusted for age, gender, race, education level, BMI, hypertension, diabetes, cigarette smoking, firearm noise exposure, occupational noise exposure, recreational noise exposure.

| Loglikelihood ratio test | 0.527 | 0.335 | 0.258 |
| --- | --- | --- | --- |
