# Supplementary material for: Association between urinary arsenic and hearing threshold shifts in adults in the United States, National Health and Nutrition Examination Survey, 2015–2016
Source: Front Public Health. 2024 Dec 18;12:1431122. doi: 10.3389/fpubh.2024.1431122 (PMC11688340; doi:10.3389/fpubh.2024.1431122)
Supplement: Supplementary file 3 [file Table_3.docx]

Table S3 The results of two-piecewise linear regression model between uDMA levels and hearing thresholds (N=1017).

| **Exposure variables** | **Low-frequency PTA** | **Speech-frequency PTA** | **High-frequency PTA** |
| --- | --- | --- | --- |
| Cut off point of uDMA | 1.57 | 2.41 | 2.78 |
| < Cut off point of uDMA | 0.59 (-1.52, 2.70), 0.5850 | 1.65 (0.41, 2.90), **0.0093** | 1.10 (-0.53, 2.74), 0.1857 |
| ≥ Cut off point of uDMA | 1.64 (-0.07, 3.36), 0.0604 | -0.43 (-4.10, 3.24), 0.8175 | -3.69 (-11.43, 4.04), 0.3499 |
| Loglikelihood ratio test | 0.527 | 0.335 | 0.258 |
